# Waist-to-Height Ratio: A Sensitive Tool for Assessing the Need for Nutritional Risk Management in Elderly Populations from Brazil

**DOI:** 10.3390/healthcare11172406

**Published:** 2023-08-28

**Authors:** Vivian C. Honorato dos Santos Carvalho, Leila B. Moreira, Vivian C. Luft, Sandra C. Fuchs

**Affiliations:** 1 Post-Graduate Program in Epidemiology, School of Medicine, Universidade Federal do Rio Grande do Sul, Rua Ramiro Barcelos, 2400, 2º Andar, Santa Cecilia, Porto Alegre 90035-003, RS, Brazil; vihonorato@hotmail.com (V.C.H.d.S.C.); vcluft@hcpa.edu.br (V.C.L.); 2 Department of Nutrition, Multidisciplinary Health Institute, Universidade Federal da Bahia, Vitória da Conquista 40170-110, BA, Brazil; 3 Post-Graduate Program in Cardiology, School of Medicine, Universidade Federal do Rio Grande do Sul, Rua Ramiro Barcelos, 2400, 2º Andar, Santa Cecilia, Porto Alegre 90035-003, RS, Brazil; lbmoreira@hcpa.edu.br

**Keywords:** BMI, waist circumference, waist-to-height ratio, obesity, abdominal obesity, sensitivity and specificity

## Abstract

Introduction: Nutritional status assessment commonly relies on body mass index (BMI), which overlooks lean mass and adipose tissue distribution. However, waist circumference (WC) and waist-to-height ratio (WHtR) provide additional insights into fat accumulation. By combining these indices, it may be possible to identify older adults needing weight management interventions. Objectives: To assess the WC and WHtR as strategies for identifying individuals requiring weight management. Methods: A cross-sectional study was conducted with 509 elderly individuals in Northeast Brazil. Weight, height, hip circumference, and waist circumference were measured, and combined with indices such as BMI WC, WHR, and WHtR to identify those who require weight management. The DeLong test compared areas under the curves using receiver operating characteristic curves and statistical significance. Sensitivity, specificity, and positive and negative predictive values were calculated to verify usefulness for clinical application. A validation sample of 599 elderly individuals from the country’s Southern region was used to confirm the results. Results: Both WC and WHtR showed adequate diagnostic accuracy with no statistically significant difference in AUCs. WHtR ≥ 0.50 had 92% sensitivity in identifying men and women requiring nutritional management. WC presented lower sensitivity but 93% specificity, useful for excluding elderly individuals from the nutritional risk category. These results were consistent in the validation sample. Conclusion: WHtR is a valuable index for screening nutritional risk management in the elderly population, applicable to men and women. Conversely, WC performs better in excluding individuals who do not need nutritional risk management.

## 1. Introduction

In both clinical practice and population-based studies, weight management has traditionally been assessed using anthropometric measures, primarily body mass index (BMI), which corrects weight for height [1]. Additionally, other simple and easily implemented anthropometric indicators, such as waist circumference (WC), waist-to-hip ratio (WHR), and waist-to-height ratio (WHtR), have been used to detect obesity [2,3,4]. However, BMI fails to consider the proportion of weight attributed to lean mass and the distribution of adipose tissue, resulting in an incomplete evaluation of excess body mass. Nevertheless, the simplicity of calculating BMI allows for its practical use in clinical settings and for characterizing cardiovascular risk [3,4].

Anthropometric indicators, such as BMI, WHR, and WC, not only provide estimates of accumulated fat but also serve as predictors of mortality [5]. However, the associations of BMI, WHR, and WC with mortality vary based on specific cutoff points for men, women, and various ethnicities [6]. Furthermore, the predictive utility of indicators like BMI and WC for assessing the risk of cardiovascular disease (CVD) over a 10-year period varies across populations [7]. Criteria have been established to identify an elevated risk of CVD based on WC, with cutoff points of ≥94 cm for men and ≥80 cm for women indicating an increased risk. Moreover, higher thresholds of ≥102 cm for men and ≥88 cm for women have been determined, indicating a substantially increased risk. These criteria were derived from population studies encompassing a wide age range.

However, the anthropometric indicators overlooked the limitations related to aging [6]. As people age, there are changes in body dimensions, including a decline in muscle mass and a redistribution of fat mass [8], particularly in women. The prevalence of overweight, obesity, and abdominal obesity differs between men and women, with women exhibiting higher rates. Moreover, these variations are influenced by the choice of indicator employed [9]. A study conducted on Caucasian individuals aged 25 to 74 years showed that WC was a valuable index for identifying individuals at risk who require nutritional management, especially when used in combination with BMI and WHR [10,11,12]. Nevertheless, the comparison between anthropometric indicators in the elderly population has not been fully evaluated [6], including the waist-to-height ratio index (WHtR) [13]. In adults, BMI and WC are valuable tools for assessing excessive body mass and estimating the associated health risks [14]. While BMI is a simple and cost-effective measurement, WC provides a more accurate assessment of abdominal fat. Combining these two indicators significantly enhances the prediction of risk for hypertension, diabetes mellitus, and metabolic syndrome [15,16]. Additionally, other indices such as the lipid accumulation product (LAP) index, body adiposity index, and neck circumference have been shown to be useful in detecting type-2 diabetes, independently of BMI and other confounding factors [17].

Overall, the existing literature supports the use of BMI, WC, and WHtR, emphasizing the importance of employing multiple measures to accurately predict obesity-related health risks in older adults [18]. We selected this approach due to its straightforwardness and the benefits it offers compared to more sophisticated methods. It has been reproduced in several studies in different populations, age ranges, and ethnicities [19]. Moreover, various studies have evaluated different cutoff points for waist circumference and BMI, providing valuable insights into their applicability for assessing nutritional risk. For instance, Misra et al. determined waist circumference cutoff points specifically for Asian Indians, highlighting the importance of considering ethnic variations [20]. Additionally, Molarius et al. examined the sensitivity of waist action levels in identifying subjects with overweight or obesity across multiple populations [21]. Several studies have also investigated the associations between anthropometric indices, such as waist-to-height ratio and cardiovascular disease (CVD) or cardiovascular risk factors [20,22]. Furthermore, an anthropometric index called the body roundness index (BRI) was developed to provide individualized weight management recommendations and predict all-cause mortality risk [23,24,25]. These additional studies contribute to the understanding of management strategies for nutritional risk. Several studies have shown that among individuals who are equally overweight or obese, those with excessive visceral adipose tissue and ectopic fat depots face a greater risk of developing metabolic complications predictive of an increased risk of CVD [26,27,28]. Based on this understanding, we aimed to investigate the association between waist circumference and the thresholds for nutritional risk that signify the need for individuals to take appropriate action. Our aim was to evaluate WC and WHtR as strategies for identifying older individuals at nutritional risk who require weight management based on a comprehensive set of anthropometric indicators, including BMI and WHR.

## 2. Materials and Methods

### 2.1. Study Design and Participants

This cross-sectional study recruited a representative sample of men and women aged 60 years or older who were receiving medical care at primary health care units in the municipality of Ilhéus, Bahia, Northeast Brazil. The participants were randomly selected from those present at the units for consultations, participating in group activities, or visiting at home. We used a stratified sampling technique to select participants from 21 out of 33 healthcare units proportionate to the presence or absence of a Family Medicine Program. Ilhéus has a population primarily composed of older individuals from diverse ethnic backgrounds, including descendants of enslaved Africans, indigenous people, and European settlers, resulting in a rich history of miscegenation. The rationale, design, and results for the model of care have been published previously [29]. To validate the findings, we replicated the analyses using a validation sample consisting of men and women aged 60 to 90 years. This sample was obtained through a random population-based selection method, representative of Porto Alegre, RS, Southern Brazil [30]. Participants were randomly chosen from 106 out of 2157 census tracts in Porto Alegre, employing a multi-stage sampling. We selected 30 households per sector and included all older individuals residing in those households. The study excluded temporary residents and domestic workers. In addition, the older population in Porto Alegre primarily consists of Caucasian individuals of European descent, and the city serves as the capital of the Rio Grande do Sul state, known for its high level of economic development. Both studies received approval from the Research Ethics Committee of the Conceição Hospital Group (GHC: 090 090/09) for the Ilhéus sample and the Research Ethics Committee of the Hospital de Clínicas de Porto Alegre (GPPG: 00-176) for the Porto Alegre sample. Written informed consent was obtained from all participants.

### 2.2. Study Variables

Data were collected using a standardized questionnaire to gather information on age, sex, years completed at school, previous medical diagnoses of comorbidities (such as hypertension, diabetes mellitus, mental disorder, chronic pain, and cardiovascular disease), and the primary health problem. Participants who reported a previous diagnosis of coronary artery disease, stroke, cardiovascular disease, heart problems, diabetes mellitus, or hypertension were classified as having cardiovascular disease (CVD).

Standardized measurements were conducted in duplicate for height (cm), weight (kg), WC (cm), and HC (cm), and the average values were used in the analysis. Height was measured using a portable stadiometer (Sanny^®®^) with participants in an upright position, barefoot, and arms along their bodies. Weight was measured using a portable scale (Techline^®®^, BAL-180-CI model) with a precision of 100 g, while individuals wore light clothing. Circumference measurements were taken in duplicate using an inelastic measuring tape. WC was measured at the midpoint between the lower costal rib and the iliac crest, and HC was measured at the level of the greater trochanter and the greatest bulge on the gluteus.

WC was categorized as increased when >94 cm for men or >80 cm for women, and as substantially increased when >102 cm for men or >88 cm for women [6]. BMI was calculated by weight (kg) divided by height (squared meters, m^2^) and was categorized as ≥25 kg/m^2^ for overweight and ≥30.0 kg/m^2^ for obesity [31]. Other variables, such as WHR [31] and WHtR [13], were also computed. WHR was categorized as ≥0.90 for men and ≥0.85 for women [6], while WHtR was considered as 0.5 for both genders.

Clinical outcomes were determined by assessing the need for nutritional risk management at levels 1 and 2, following the methodology suggested by Lean, Han, and Morrinson [10]. This approach involved the combination of indicators such as BMI and WHR. Please refer to Table 1 for the specific criteria used to determine the different levels of nutritional risk and to define the corresponding management strategies [10].

### 2.3. Research Team

Research assistants conducted standardized evaluations under the supervision of a certified researcher. The team consisted of undergraduate students from the Department of Nutrition, and interviews were conducted using a previously tested standardized questionnaire. To ensure the reliability of the data collection, a pilot study was carried out with elderly individuals who were not included in the final sample. For the validation sample, the research team comprised certified coordinators, supervisors, and interviewers who conducted the interviews in participants’ homes using a similar standardized questionnaire

### 2.4. Sample Size Calculation and Statistical Analysis

The sample size was calculated for the primary objective [29], using the Epidat program version 3.1, Xunta de Galicia, PAHO/WHO. In this analysis, it was estimated that one of the anthropometric indicators would have 85–95% sensitivity to detect obesity, estimated at 32%, with a confidence interval (CI) of 95% and power of 80%. The calculation was expanded by 15% to preserve the statistical power in case of losses, which resulted in a sample of 508 elderly individuals.

The receiver operating characteristic (ROC) curve was utilized to evaluate the anthropometric indices, namely WC and WHtR, in relation to the clinical outcomes of nutritional risk levels 1 and 2. These risk levels were calculated separately for each sex. The accuracy of the anthropometric indicators in determining older individuals at nutritional risk was described using the area under the curve (AUC) along with a 95% confidence interval (CI). Additionally, sensitivity, specificity, and positive and negative predictive values (PPV and NPV) were calculated for the evaluated cutoff points [32].

The characteristics of the sample are presented as means and standard deviations (mean ± SD) for quantitative variables, and as frequencies and percentages (n and %) for categorical variables. These descriptive statistics are provided separately for men and women. To assess the normal distribution of the quantitative variables, the Kolmogorov–Smirnov test was employed. Proportions were compared using Pearson’s χ^2^ test, while differences between means were evaluated using Student’s *t* test. Statistical analyses were performed using the Statistical Package for the Social Sciences version 18.0 (SPSS Inc., Chicago, IL, USA), and the Epidat program was used to compare ROC curves using the DeLong test.

## 3. Results

Out of the 511 eligible individuals in the Northeast, 509 agreed to participate in the study. The findings showed that around 65% of women and 49% of men had excess weight. Moreover, 82% of the participants had a WHR equal to or greater than 0.85, while 85% had a WHR of 0.90 or higher. Table 2 presents the characteristics of the participants according to sex. The average age of the participants was 72.8 ± 8.2 years, with the majority being women. Furthermore, almost half of the participants had not completed the first year of elementary school. On average, women had higher BMI and WHtR, whereas men exhibited a higher WHR index.

Table 3 presents the distribution of participants at nutritional risk levels 1 and 2, categorized by WC and WHtR cutoff points, for older men and women. This analysis investigated the prevalence of enlarged WC among two groups within the elderly population: individuals with nutritional risk level 1, defined as having a BMI ≥ 25 kg/m^2^, and those with BMI < 25 kg/m^2^ but high WHR. The study also examined the prevalence of enlarged waist-to-height ratio (WHtR) for these two groups. Overall, the findings indicated that older men and women with BMI ≥ 25 kg/m^2^ had a high prevalence of enlarged WC, which reinforces the well-established association between increased BMI and abdominal obesity. Additionally, the analysis showed similar results for WHtR of 0.5 or higher. These findings also highlighted that older men and women with a normal BMI but increased WHR exhibited central obesity, although men had a relatively lower prevalence of abnormal WC. However, individuals with this phenotype did not show enlarged WHtR.

Table 3 also presents these associations for individuals with nutritional risk level 2: those with BMI ≥ 30 kg/m^2^ and those with BMI < 30 kg/m^2^ but high WHR. Older men and women with obesity had a high prevalence of enlarged WC and WHtR. Furthermore, older individuals with a non-obese BMI but enlarged WHR also showed a high prevalence of abdominal obesity. Similar patterns were observed when examining the association with WHtR of 0.5 or higher. These findings also emphasize that older men and women with a non-obese BMI can still have a central deposit of fat, making them candidates for weight management interventions.

Table 4 presents the diagnostic properties of anthropometric indices for screening different levels of nutritional risk. When detecting level 1 nutritional risk in men, an increased WC exhibited low sensitivity, whereas a normal waist measurement excluded it. Conversely, WHtR showed high sensitivity and specificity. For women, the WHtR test had high sensitivity and specificity. Compared to WC, a WHtR of 0.50 or higher could be used as a diagnostic test to identify individuals with a nutritional risk that requires management, regardless of gender. Concerning nutritional risk level 2, WC displayed very low sensitivity but high specificity. Conversely, WHtR remained a sensitive test, albeit with the drawback of lower specificity.

**Figure 1:**
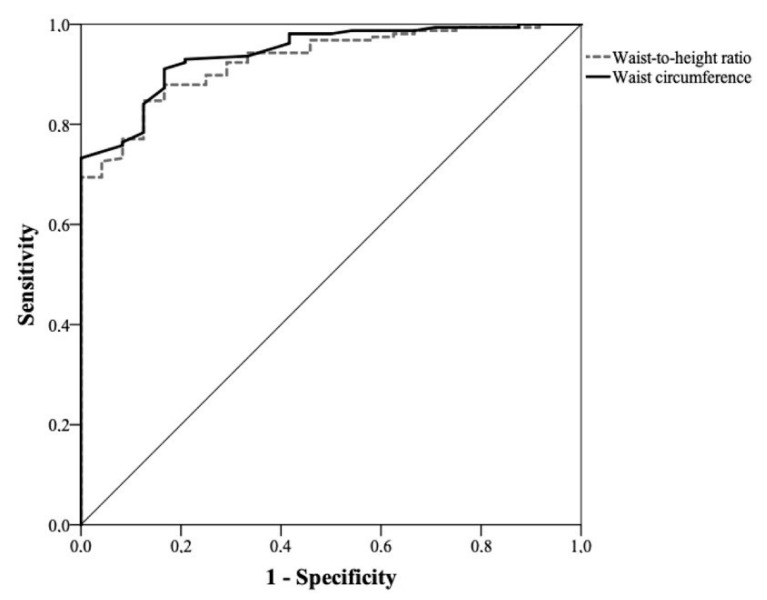
ROC curve of waist circumference and waist-to-height ratio for assessing nutritional risk level 1 in men (AUC for WC: 0.94 (0.90–0.98) and WHtR: 0.93 (0.89–0.97); *p* = 0.6).

**Figure 2:**
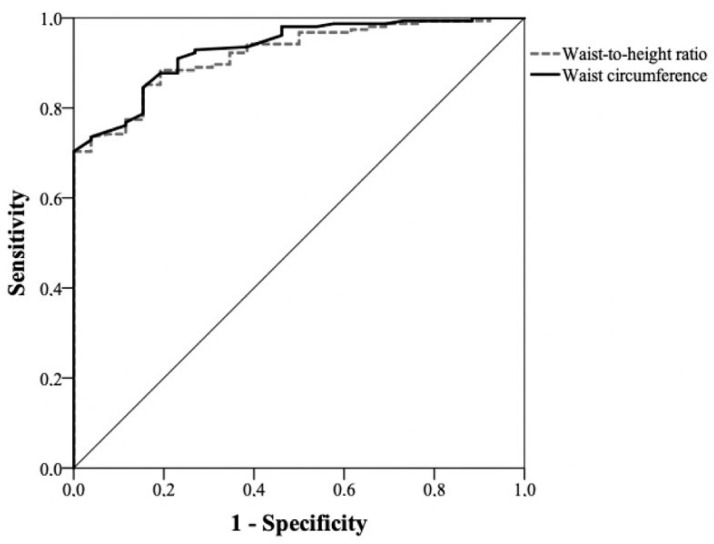
ROC curve of waist circumference and waist-to-height ratio for assessing nutritional risk level 2 in men (AUC for WC: 0.93 (0.89–0.97) and WHtR: 0.92 (0.88–0.96); *p* = 0.7).

**Figure 3:**
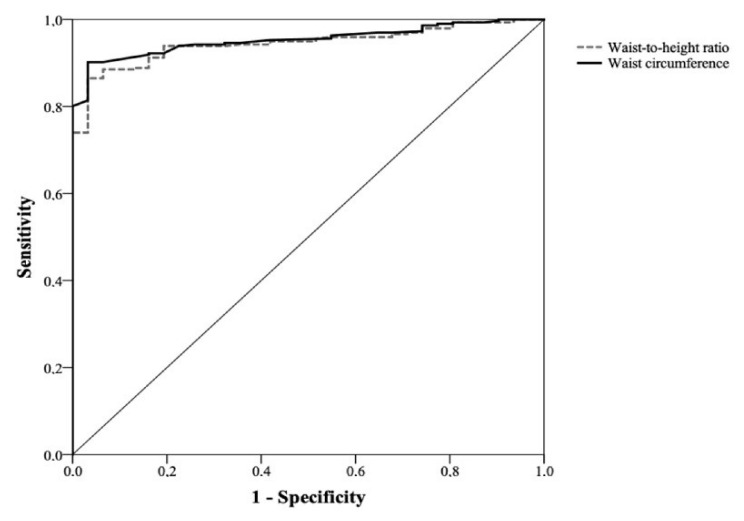
ROC curve of waist circumference (WC) and waist-to-height ratio (WHtR) for assessing nutritional risk level 1 in women (AUC for WC: 0.95 (0.93–0.98) and WHtR: 0.94 (0.92–0.97); *p* = 0.6).

**Figure 4:**
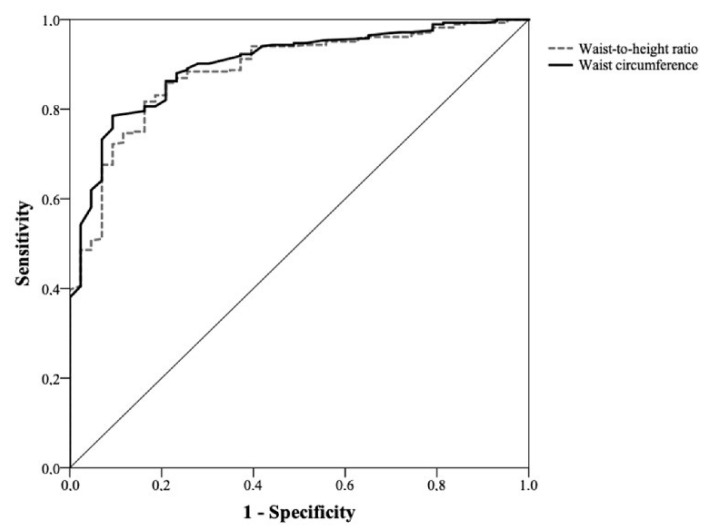
ROC curve of waist circumference (WC) and waist-to-height ratio (WHtR) for assessing nutritional risk level 2 in women (AUC for WC: 0.90 (0.86–0.94) and WHtR: 0.89 (0.84–0.93); *p* = 0.7).

The comparison between the ROC curves of WC and WHtR did not show statistically significant differences, with very similar areas under the curve. Figure 1 and Figure 2 depict the ROC curves for nutritional risk levels 1 and 2 in men, respectively. The comparison of the ROC curves between WC and WHtR showed very similar areas under the curve, indicating no statistically significant differences between them. Similarly, Figure 3 and Figure 4 depict the ROC curves for nutritional risk levels 1 and 2 in women, respectively. The ROC curves of WC and WHtR also demonstrated comparable areas under the curves, with no statistically significant differences observed.

Table 5 displays higher sensitivity of WC in women than men in detecting level 1 nutritional risk. On the other hand, WHtR exhibits greater sensitivity and positive predictive value (PPV) for both men and women. Consequently, over 96% of elderly individuals classified at a nutritional risk level 1 would have a WHtR value equal to or greater than 0.50.

In detecting nutritional risk level 2, WHtR was the most sensitive indicator for clinical use in both men and women. Comparing the performance of WC and WHtR indices in determining nutritional risk levels in both sample groups, WHtR ≥ 0.50 displayed high sensitivity and PPV in identifying nutritional risk among elderly individuals. On the other hand, having a normal WC was clinically relevant in excluding nutritional risk for both men and women in the Northeast sample but only for men in the validation sample.

Table 6 presents the characteristics of the 599 participants from the validation sample. Compared to the Northeast sample, the older population in the validation sample had a lower proportion of illiterate individuals and a lower reported prevalence of cardiovascular disease (CVD) as the leading health issue. Statistically significant differences were observed between older men and women regarding BMI, WC, and WHtR. On average, women had higher BMI but lower WC and WHtR than men.

Table 7 presents the prevalence of older individuals in the validation sample who require nutritional risk management based on WC and WHR. Among those with excess weight or a BMI < 25 kg/m^2^ but a higher WHR, there was a higher prevalence of abnormal WHtR compared to WC. The validation sample yielded similar findings to the Northeast sample, indicating that more individuals were classified based on BMI alone rather than the normal BMI and high WHR combination.

## 4. Discussion

Our results differed from those obtained in a population sample of European individuals aged 25 to 74 years for waist circumference [10]. In the previous study, WC could be used in health promotion programs to identify individuals who would be eligible for weight control. The present study examined anthropometric indices for detecting nutritional risk levels requiring management in elderly individuals. When compared to the combination of BMI and WHR, both WC and WHtR indices demonstrated adequate AUC values, indicating useful diagnostic accuracy for clinical applications. The WC and WHtR indices proved effective in screening and determining levels of nutritional risk in older men and women. WHtR ≥ 0.50 emerged as the most sensitive indicator for identifying individuals at nutritional risk, while a normal WC exhibited the highest specificity for excluding risk. Our findings contrasted with a previous study on a population sample of European individuals aged 25 to 74 years, specifically regarding waist circumference (WC) [10]. In that study, WC was suggested as a valuable tool for health promotion programs, enabling the identification of individuals suitable for weight control interventions.

The study’s findings showed a high prevalence of excess weight, predominantly among women, accompanied by an elevated WHR in most participants. Waist-to-height ratio emerged as a sensitive indicator for detecting nutritional risk, particularly at level 2, indicating central fat accumulation. Although WC exhibited low sensitivity, it remained clinically relevant in excluding nutritional risk. The comparison of ROC curves between WC and WHtR presented similar performance without any statistically significant differences. Furthermore, the validation sample yielded results comparable to those of the Northeast sample, underscoring the generalizability of the findings. These findings highlight the significance of WHtR as a sensitive tool for nutritional risk assessment in elderly populations and emphasize the necessity for targeted interventions tailored to regional variations in nutritional profiles.

Our study provided insights into the use of anthropometric indices for identifying nutritional risk in the older population. While we acknowledge that anthropometric indices can vary according to ethnicity, our study included populations with diverse ethnic backgrounds in both the study population from Ilhéus and the validation sample. By including populations with varying ethnic composition to test the same hypothesis and evaluate the consistency of the results across different populations, our study provided insights that are applicable to a broader range of individuals in Brazil.

However, it is essential to recognize that further research focusing on ethnicity and its influence on anthropometric indices would enhance our understanding of these variations and their implications for nutritional risk management. We acknowledge that the lack of analysis by specific ethnic groups is a limitation of our study. Future investigations that include a more comprehensive analysis by ethnicity would provide insights into the potential variations and considerations for nutritional risk management in older populations.

In addition to the disparity in the proportions of elderly individuals and the prevalence of overweight and abnormal WHR observed among the studied populations, the contrasting results can be attributed to variations in lifestyle and genetic profiles. The prominence of WC as a performance indicator has been established through analyses of clinical outcomes, including multi-morbidities [33], cardiovascular risk factors [34,35], non-communicable diseases [36], and cardiovascular outcomes [13]. However, certain studies have shown advantages for WHtR as an anthropometric index [2,37]. WHtR can be utilized with a single cutoff point, while WC involves the use of four cutoff points for men and women. Furthermore, the consistent results obtained from both the Northeast and validation samples regarding nutritional risk screening using WHtR strengthens its use in clinical practice. Consequently, a simple and unified message is appropriate for both men and women: “waist circumference should not exceed half of the height”. These findings provide valuable insights for promoting nutritional health among elderly individuals residing in the Southern and Northeast regions of Brazil.

To ensure a comprehensive interpretation of the results, it is important to acknowledge the limitations associated with the primary outcome measures used to define nutritional risk. Relying solely on BMI and WHR may overlook important factors such as the proportion of weight attributed to lean mass and the distribution of adipose tissue. This incomplete evaluation of excess body mass can be improved by considering a central obesity index that takes into account the distribution of adipose tissue [17]. By incorporating such measures, a more accurate assessment of excess body mass can be achieved, leading to a more nuanced understanding of the relationship between body composition and health outcomes in the context of nutritional risk management.

The existing literature provides support for the effectiveness of indicators such as BMI and WC in assessing cardiovascular risk factors [34,35,36]. While we recognize the limitations of BMI as a measure of overall obesity, we still believe it can be a valuable tool [38]. In our study, we utilized a combination of various anthropometric indicators to screen for nutritional risk at levels 1 and 2, allowing us to assess both general and central obesity and enhance the sensitivity of our testing approach. The use of a combined indicator is particularly relevant due to its clinical applicability and feasibility in providing nutritional recommendations. It is worth noting that anthropometric indicators can vary with age, and older individuals tend to have lower values compared to younger ones [39]. A limitation of our study is the absence of a specific analysis by ethnic groups. While our study included a population with diverse ethnic backgrounds, we did not perform an examination of the variations in anthropometric indices and nutritional risk levels among specific ethnic groups. This limitation hinders a comprehensive understanding of the potential influence of ethnicity on these measures. Future studies should prioritize a thorough analysis by ethnic groups to unravel the variations and implications for nutritional risk management among different ethnic older populations, providing a more nuanced understanding of the interplay between ethnicity and anthropometric indices. Thus, further studies are needed to explore the use of anthropometric indicators in older populations. However, although there are differences between men and women and among populations, age-related variations tend to be similar in older individuals from different nationalities [39]. Other combinations of anthropometric indicators could be used to determine health risks and identify hypertension [40]. The choice of a WHtR cutoff point ≥ 0.50 was based on a previous meta-analysis that included individuals of different ethnicities and ages from 14 countries [41]; however, there are higher cutoff points [13,42]. Moreover, the cutoff points of WHtR seemed to increase with age in women, but not in men [43].

The distribution of adiposity varies between genders and can be accentuated with aging, resulting in muscle mass loss, redistribution, and a significant reduction in fat mass, particularly in women [8]. Despite gender and population differences, age-related variations in anthropometric indicators tend to be similar among elderly individuals of various nationalities [39]. Alternative combinations of anthropometric indicators can be investigated to assess health risks and identify hypertension, drawing insights from studies conducted on adult populations for result comparisons [10]. Therefore, if the selection of the cutoff point introduced a measurement bias, it was unintentional and necessitates further examination. The superiority of WHtR compared to WC regarding sensitivity may result from the cutoff point used for the latter. However, the cutoff points were established based on recommendations [6,13] that may underrepresent the best cutoff points for elderly individuals. Considering the lack of statistical significance for diagnostic accuracy between WHtR and WC distributions, further studies evaluating other cutoff points for elderly individuals are warranted.

In conclusion, the assessment of nutritional risk at levels of action using the WHtR is consistent between men and women, allowing its use for decision-making in the nutritional management of the elderly population. In addition, WHtR can be used as a screening method in the clinical setting and enables a direct educational message for use in different healthcare practice scenarios: “your waist cannot be greater than half of your height”.

## Figures and Tables

**Table 1:** Definition of clinical outcomes based on BMI and WHR for men and women.

	Nutritional Risk Level 1 *	
	BMI (kg/m^2^)	BMI (kg/m^2^) and WHR
Men	≥25 or <25 and WHR ≥ 0.90	
Women	≥25 or <25 and WHR ≥ 0.85	
	Nutritional risk level 2 *	
	BMI (kg/m^2^)	BMI (kg/m^2^) and WHR
Men	≥30 or <30 and WHR ≥ 0.90	
Women	≥30 or <30 and WHR ≥ 0.85	

* Adapted from Lean, Han, and Morrinson [10].

**Table 2:** Characteristics of the elderly population according to sex [mean ± DP * or n (%) **] (Ilhéus, BA, Brazil).

	Total n = 509	Men n = 182	Women n = 327	*p*-Value
Age (years)	72.8 ± 8.2	73.0 ± 8.2	72.6 ± 8.2	0.7
Age range (years)	60.1–103.4	60.3–93.6	60.1–103.4	
Schooling (years)				1.0
0	241(47.3)	85 (46.7)	156 (47.7)	
1 to 4	190 (37.3)	68 (37.4)	122 (37.3)	
≥5	78 (15.3)	29 (15.9)	49 (15.0)	
Body mass index (kg/m^2^)	26.6 ± 5.3	25.3 ± 4.2	27.3 ± 5.7	<0.001
Waist circumference (cm)	90.3 ± 13.4	91.1 ± 12.8	89.9 ± 13.7	0.4
Waist-hip ratio	0.94 ± 0.09	0.98 ± 0.10	0.92 ± 0.09	<0.001
Waist-to-height ratio	0.58 ± 0.09	0.56 ± 0.07	0.59 ± 0.09	<0.001
Hypertension	144 (28.3)	45 (24.9)	99 (30.2)	0.2
Diabetes mellitus	67 (13.2)	18 (9.9)	49 (14.9)	0.12
Mental disorder	9 (1.8)	5 (2.8)	4 (1.2)	0.2
Chronic pain	126 (24.9)	47 (26.0)	79 (24.3)	0.7
Main health problem: CVD	204 (40.1)	63 (34.6)	141 (43.1)	0.06

* Student’s *t* test. ** χ^2^ test.

**Table 3:** Elderly population at greater nutritional risk requiring management according to WC and WHtR, for men and women (n and %) (Ilhéus, BA, Brazil).

	Nutritional Risk Level 1	
	BMI ≥ 25 kg/m^2^	BMI < 25 kg/m^2^ and High WHR
Men	n = 89	n = 68
WC > 94 cm	60 (67.4) *	14 (20.6) *
WHtR ≥ 0.5	87 (97.8) *	58 (85.3) ^ƒ^
Women	n = 212	n = 84
WC > 80 cm	201 (94.8) *	43 (51.2) *
WHtR ≥ 0.5	207 (97.6) *	68 (81.0) ^ƒƒ^
Total	n = 301	n = 152
WC > 94/>80 cm	261 (86.7) *	57 (37.5) *
WHtR ≥ 0.5	294 (97.7) *	126 (82.9) ^ƒƒƒ^
	Nutritional Risk Level 2	
	BMI ≥ 30 kg/m^2^	BMI < 30 kg/m^2^ and high WHR
Men	n = 26	n = 129
WC > 102 cm	17 (65.4) *	18 (14.0) ****
WHtR ≥ 0.5	26 (100.0) **	117 (90.7) *
Women	n = 96	n = 188
WC > 88 cm	90 (93.8) *	92 (48.9) *
WHtR ≥ 0.5	96 (100.0) *	168 (89.4) ***
Total	n = 122	n = 317
WC > 102/> 88 cm	107 (87.7) *	110 (34.7) *
WHtR ≥ 0.5	122 (100.0) *	285 (89.9) *

High WHR ≥ 0.90 for men and ≥0.85 for women. * χ^2^ test; *p* < 0.001. ** χ^2^ test; *p* = 0.02. *** χ^2^ test; *p* = 0.04. **** χ^2^ test; *p* = 0.005. ^ƒ^ χ^2^ test; *p* = 0.6. ^ƒƒ^ χ^2^ test; *p* = 0.13. ^ƒƒƒ^ χ^2^ test; *p* = 0.4.

**Table 4:** Diagnostic properties of anthropometric indices to detect levels of nutritional risk requiring management (Ilhéus, BA, Brazil).

	Cutoff Point	Sensitivity (95% CI)	Specificity (95% CI)	PPV (95% CI)	NPV (95% CI)
Level 1: BMI ≥ 25 kg/m^2^ or BMI < 25 kg/m^2^ and high WHR					
Men	WC >94 cm	46.8 (38.7–54.9)	100.0 (97.9–100.0)	100.0 (99.3–100.0)	22.4 (14.1–30.8)
	WHtR ≥ 0.50	91.8 (81.2–96.4)	70.8 (50.6–91.1)	95.4 (91.7–99.1)	56.7 (37.3–76.1)
Women	WC >80 cm	82.4 (77.9–86.9)	96.8 (88.9–100)	99.6 (98.6–100.0)	36.6 (25.6–47.6)
	WHtR ≥ 0.50	92.9 (89.8–96.0)	80.7 (65.1–96.2)	97.9 (96.0–99.7)	54.4 (38.9–69.8)
Level 2: BMI ≥ 30 kg/m^2^ or BMI < 30 kg/m^2^ and high WHR					
Men	WC > 102 cm	22.6 (15.7–29.5)	100.0 (98.1–100.0)	100.0 (98.6–100.0)	17.8 (11.3–24.4)
	WHtR ≥ 0.50	91.7 (87.0–96.3)	65.4 (45.2–85.6)	94.1 (90.0–98.2)	56.7 (37.3–76.1)
Women	WC >88 cm	64.1 (58.3–69.8)	93.0 (84.3–100.0)	98.4 (96.3–100.0)	28.2 (20.4–35.9)
	WHtR ≥ 0.50	93.0 (89.8–96.1)	60.5 (44.7–76.2)	94.0 (91.0–96.9)	56.5 (41.1–71.9)

**Table 5:** Diagnostic properties and cutoff points of anthropometric indicators in relation to nutritional risk in the validation sample.

	Cutoff Point	Sensitivity (95% CI)	Specificity (95% CI)	PPV (95% CI)	NPV (95% CI)
Level 1: BMI ≥ 25 kg/m^2^ or BMI < 25 kg/m^2^ and high WHR					
Men	WC >94 cm	76.2 (69.5–82.8)	100.0 (96.4–100.0)	100.0 (99.6–100.0)	25.5 (13.0–37.9)
	WHtR ≥0.50	97.7 (95.1–100.0)	64.3 (35.6–93.0)	97.1 (94.3–99.9)	69.2 (40.3–98.2)
Women	WC >80 cm	93.3 (90.5–96.1)	81.0 (70.5–91.4)	96.4 (94.2–98.5)	68.9 (57.7–80.1)
	WHtR ≥ 0.50	96.2 (94.0–98.4)	68.3 (56.0–80.5)	94.3 (91.7–96.8)	76.8 (64.8–88.7)
Level 2: BMI ≥ 30 kg/m^2^ or BMI < 30 kg/m^2^ and high WHR					
Men	WC >102 cm	43.1 (35.3–50.9)	100.0 (97.4–100.0)	100.0 (99.3–100.0)	16.7 (9.4–24.0)
	WHtR ≥ 0.50	97.6 (95.0–100.0)	47.4 (22.3–72.5)	94.2 (90.5–98.0)	69.2 (40.3–98.2)
Women	WC >88 cm	78.0 (73.1–82.9)	90.1 (84.1–96.1)	95.4 (92.6–98.3)	60.6 (52.9–68.4)
	WHtR ≥ 0.50	98.0 (96.2–99.7)	45.0 (35.3–54.8)	82.5 (78.3–86.6)	89.3 (80.3–98.3)

**Table 6:** Characteristics of the elderly population in the validation sample according to sex [mean ± SD * or n (%) **] (Porto Alegre, RS, Brazil).

	Total n = 599	Men n = 187	Women n = 412	*p*-Value
Age (years)	70.7 ± 7.2	70.3 ± 6.6	70.9 ± 7.6	0.3
Schooling (years)				<0.001
0	49 (8.2)	5 (2.7)	44 (10.7)	
1 to 4	155 (25.9)	39 (20.9)	116 (28.2)	
≥5	394 (65.9)	143 (76.5)	251 (61.1)	
Body mass index (kg/m^2^)	28.0 ± 5.0	27.2 ± 4.2	28.4 ± 5.2	0.007
Waist circumference (cm)	94.4 ± 13.5	99.7 ± 11.2	91.9 ± 13.7	<0.001
Waist-hip ratio	0.91 ± 0.10	0.98 ± 0.07	0.89 ± 0.11	<0.001
Waist-to-height ratio	0.59 ± 0.08	0.59 ± 0.06	0.59 ± 0.09	0.7
Main health problem: CVD	58 (8.3)	37 (9.0)	21 (11.2)	0.4

* Student’s *t* test. ** χ^2^ test.

**Table 7:** Elderly population at greater nutritional risk requiring management according to WC and WHtR, for men and women [(n and %) *] (Porto Alegre, RS, Porto Alegre).

	Nutritional Risk Level 1	
	BMI ≥ 25 kg/m^2^	BMI < 25 kg/m^2^ and High WHR
Men	n = 132	n = 40
WC > 94 cm	118 (89.4)	13 (32.5)
WHtR ≥ 0.5	132 (100.0)	36 (90.0)
Women	n = 294	n = 44
WC > 80 cm	280 (95.2)	36 (81.8)
WHtR ≥ 0.5	286 (97.3)	40 (90.9)
Total	n = 426	n = 84
WC > 94/>80 cm	398 (93.4)	49 (58.3)
WHtR ≥ 0.5	418 (98.1)	76 (90.5)
	Nutritional Risk Level 2	
	BMI ≥ 30 kg/m^2^	BMI < 30 kg/m^2^ and high WHR
Men	n = 43	n = 124
WC > 102 cm	41 (95.3)	31 (25.0)
WHtR ≥ 0.5	43 (100.0)	120 (96.8)
Women	n = 138	n = 152
WC > 88 cm	136 (98.6)	92 (60.5)
WHtR ≥ 0.5	138 (100.0)	147 (96.7)
Total	n = 181	n = 276
WC > 102/>88 cm	177 (97.8)	123 (44.6)
WHtR ≥ 0.5	181 (100.0)	267 (96.7)

High WHR ≥ 0.90 for men and ≥ 0.85 for women. * χ^2^ test.

## Data Availability

The original data were presented in the article, but can be made available upon request.
